# Clinically Relevant Insulin Degludec and Its Interaction with Polysaccharides: A Biophysical Examination

**DOI:** 10.3390/polym12020390

**Published:** 2020-02-09

**Authors:** Shahwar Imran Jiwani, Sha Huang, Oritsegidenene Beji, Philemon Gyasi-Antwi, Richard B. Gillis, Gary G. Adams

**Affiliations:** 1 Queen’s Medical Centre, Faculty of Medicine and Health Sciences, University of Nottingham, Clifton Boulevard, Nottingham NG7 2HA, UK; bwzpg3@exmail.nottingham.ac.uk (P.G.-A.); richard.gillis@nottingham.ac.uk (R.B.G.); 2 School of Biosciences, Sutton Bonington Campus, University of Nottingham, Sutton Bonington, Leicestershire LE12 5RD, UK; stxsh16@nottingham.ac.uk (S.H.); nenebeji@gmail.com (O.B.)

**Keywords:** insulin degludec, xanthan, alginate, interactions, complexations

## Abstract

Protein polysaccharide complexes have been widely studied for multiple industrial applications and are popular due to their biocompatibility. Insulin degludec, an analogue of human insulin, exists as di-hexamer in pharmaceutical formulations and has the potential to form long multi-hexamers in physiological environment, which dissociate into monomers to bind with receptors on the cell membrane. This study involved complexation of two negatively charged bio-polymers xanthan and alginate with clinically-relevant insulin degludec (PIC). The polymeric complexations and interactions were investigated using biophysical methods. Intrinsic viscosity [η] and particle size distribution (PSD) of PIC increased significantly with an increase in temperature, contrary to the individual components indicating possible interactions. [η] trend was X > XA > PIC > A > IDeg. PSD trend was X > A > IDeg > XA > PIC. Zeta (ζ)- potential (with general trend of IDeg < A < XA < X ≈ PIC) revealed stable interaction at lower temperature which gradually changed with an increase in temperature. Likewise, sedimentation velocity indicated stable complexation at lower temperature. With an increase in time and temperature, changes in the number of peaks and area under curve were observed for PIC. Conclusively, stable complexation occurred among the three polymers at 4 °C and 18 °C and the complex dissociated at 37 °C. Therefore, the complex has the potential to be used as a drug delivery vehicle.

## 1. Introduction

Protein and polysaccharides have been widely used for a variety of biotechnological and pharmaceutical applications such as medical scaffolding and implants, drug delivery and inert diluent for drug and wound dressings [1,2,3] because of their high biocompatibility and biodegradability [4].

Insulin degludec (IDeg), like many other clinical insulins in use, is composed of two chains A and B. However, the human amino-acid sequence is altered through the threonine residue at position 30 on B-chain being removed and replaced by an acylated ligand containing a glutamic acid spacer. This spacer is attached to ε-amino group of Lys at B29 with hexadecanoic acid. IDeg exists in di-hexameric form where the two-hexamer units are linked together by Zn^2+^ [5,6]. These changes are made to increase the pharmacokinetic profile of insulin by forming large supra-molecular structure as well as binding with albumin in the blood stream.

Alginates (A) are linear polymers of 1,4-linked β-D-mannuronic acid residues and 1,4-linked α-L-guluronic residues, containing homo-polymeric sequences [7]. Xanthan (X) is composed of D-glucosyl, D-mannosyl, and D-glucuronyl acid residues in a 2:2:1 molar ratio and variable proportions of O-acetyl and pyruvyl residues. Its main chain consists of β-D-glucose units linked at the 1 and 4 positions. Side-chains consist of a tri-saccharide composed of mannose (β-1,4) glucuronic acid and (β-1,2) mannose, attached to alternate glucose residues in the backbone by α-1,3 linkages [8,9]. 

Xanthan gum and alginate complexes (XA) have been studied previously [10] and used for functional foods [11], tissue engineering [3,12,13] and drug delivery. As negatively charged polymers, xanthan [14,15,16,17] and alginate [18,19,20] have been used separately, and in combination with positively charged polymers such as chitosan, as potential vehicles for insulin delivery. 

Interactions among different types of protein–polysaccharide complexes (PPCs) have been previously investigated using a variety of methods [21,22]. The formation and dissociation of PPCs and their solubility depend on many factors such as surface charge, pH, temperature and ionic strength of the solvent [23]. These factors greatly influence non-covalent interactions such as electrostatic, H-bonding, hydrophobic, and steric interactions, as well as covalent interactions [24]. Conjugation of polysaccharides with therapeutic proteins is a well-established phenomenon [25]. In particular, complexes of insulin with polysaccharides have been studied to identify the possibilities for prolonged and extended half-life [26,27]. In this present study, we aimed to investigate, for the first time, whether there is any interaction(s) and complex formation between xanthan, alginate and Insulin degludec. 

## 2. Methodology

### 2.1. Materials

Insulin degludec (IDeg, 3.6 mg/mL–100 units/mL), (Tresiba, Novo Nordisk) was provided by InDependent Diabetes Trust (Northampton, UK). Disodium hydrogen phosphate dodecahydrate (Na_2_HPO_4_.12H_2_O), potassium phosphate (KH_2_PO_4_), sodium chloride (NaCl) were purchased from Fisher scientific (Loughborough, UK), xanthan gum (X) and sodium Alginate (A) were purchased from Sigma Aldrich (Gillingham, UK). All chemicals were of analytical grade. Molecular weight and hydrodynamic radii of all individual components are included in supplementary material (Supplementary Table S1). Information about degree of substitution was not provided by the manufacturer. 

### 2.2. Preparation of Polymeric-Insulin Complex (PIC) for Stability Test Using Hydrodynamical Analysis

Three sets of samples were prepared. The first set had single components, which were xanthan (X), alginate (A), and IDeg (1 mg/mL, 50 mM PBS) to give baseline results. The second set had two-component systems which were X + A, X + IDeg, A + IDeg (1:1 *v*/*v*, 1 mg/mL, and 50 mM PBS). 

For PIC preparation, the stock solution of polysaccharides was prepared by mixing X and A in equal amount by mass to obtain final concentration of 17 mg/mL in 50 mM PBS (Phosphate Buffered Saline) at pH 7.0. Concentration was confirmed using a refractometer, ATAGO DD7 (ATAGO, Tokyo, Japan) with a refractive index increment (dn/dc) of 0.154 mL/g [28]. The stock solution was stored at 4 °C. 1000 µL of solution was prepared by adding 200 µL of 3.6 mg/mL IDeg to 800 µL of 2.0 mg/mL XA solution, resulting in an 8:8:7.2 mass ratio. Final concentrations of the components were adjusted to 1 mg/mL using 50 mM PBS, pH 7.0. All samples, prepared with each set of components, were analysed for their stability incubated at three different temperatures (4, 18 and 37 °C) for 1, 7 and 14 days. 

### 2.3. Intrinsic Viscosity 

Viscosity measurements were performed using an Ostwald viscometer suspended in a water bath equilibrated to (20.0 ± 0.05) °C. Sample dilutions were prepared in the concentration range of 0.1–1.75 mg/mL. The flow times were recorded using an automated timer. From the solution/solvent flow-time ratio (t/t_0_) the relative viscosity (η_rel_) was obtained. Intrinsic viscosity was calculated using a polynomial analysis of specific viscosity (η_sp_ ≡ η_rel_ − 1) against concentration, as obtained using the following equation [29].

η_sp_ = [η]c + [η] ^2^ c^2^ (1)

### 2.4. Particle Size Distribution (PSD)

1mg/mL of samples were analysed for size distribution analysis using a Zetasizer NanoZS (Malvern Instruments, UK) with Zetasizer Software v6.0. Analysis was performed at 173° scattering angle at 4 °C, 18 °C and 37 °C using disposable polystyrene cuvettes. For each sample 6 measurements (with 10 runs each) were performed and Z-average radius was used to calculate PSD. Equilibration time was at least 180 s for each measurement. 

### 2.5. Zeta Potential (ζ) Analysis 

ζ analysis was carried out using a Zetasizer NanoZS (Malvern Instruments, Malvern, UK) with Zetasizer Software v6.0. Samples were loaded into the dip cell. Working dilutions were prepared in deionised water for all samples (insulin, alginate and xanthan) in the range of 0.1–2 mg/mL. Measurements were taken at 8 different temperatures from 5 °C to 40 °C and cuvettes were allowed to equilibrate to each temperature for at least 2 min.

### 2.6. Sedimentation Velocity 

A Beckman Optima XL-I analytical ultracentrifuge was used to perform sedimentation velocity experiments. The loading amount of sample and buffer (50 mM PBS, pH 7.0) solutions was 400 µL where each sample was of 1 mg/mL. Solutions were centrifuged at 40,000 rpm (129,000× *g*) at a temperature of 20.0 ± 0.1 °C for 12 h. Data were analysed using a least square Gaussian (ls-g*(s)) method in SEDFIT software [30]. Monte-Carlo approach was used to estimate standard errors. 

### 2.7. Statistical Analysis

Statistical significance of the difference between intrinsic viscosities at 20 °C and 37 °C was assessed using a two-tailed Z-test. ANOVA was used to assess significance of PSD against temperature. 

A multiple regression general linear model (MANOVA) was performed on the zeta potential dataset using SPSS v23 (IBM). Controlled variables included sample, temperature and concentration. Data were checked for obvious anomalies, but none were observed. Type IV Sum of Squares was used to account for small differences in n for different subsets. A post-hoc Tukey HSD test was performed to assess groupings between samples.

In all cases, *p* values were considered significant at <0.05. 

## 3. Results and Discussion 

### 3.1. Intrinsic Viscosity

**Figure 1:**
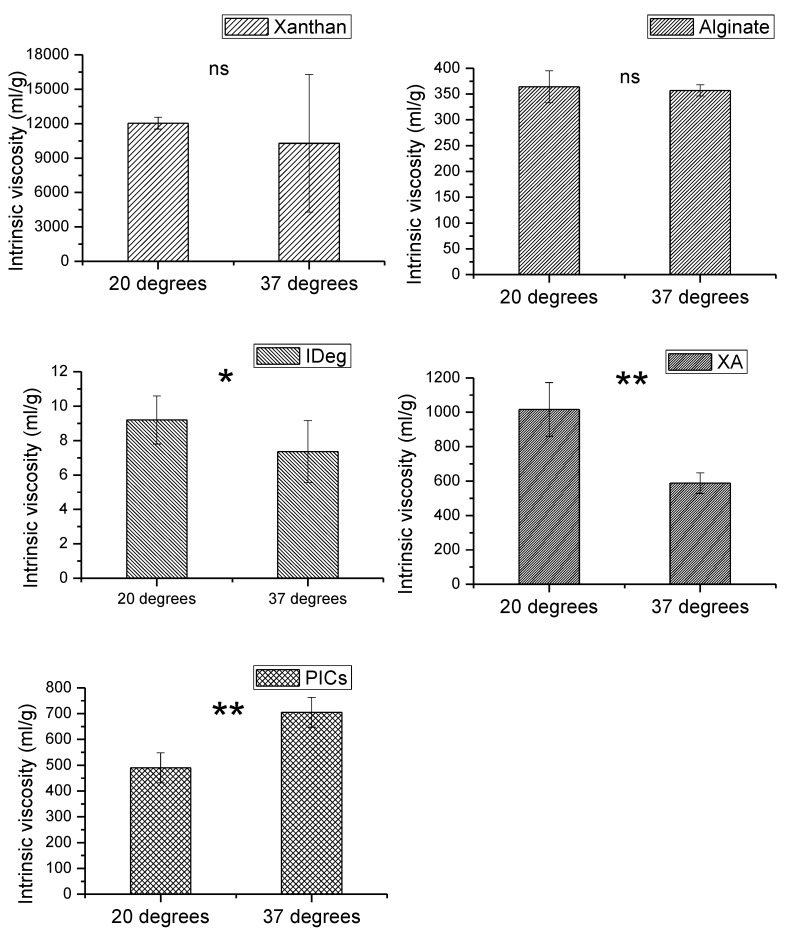
Intrinsic viscosity of xanthan (X), Alginate (A), degludec (IDeg), mixture of xanthan and Alginate (XA) and PIC measured at 20 °C and 37 °C. Data points represent SER, *n* = 3). ** (significant; *p* ≤ 10^−13^), *(significant; *p* ≤ 0.05), ns = non-significant.

Intrinsic viscosity of the individual components (X, A and IDeg), binary system (XA) and the complex (PIC) was measured using a series of concentrations (mention the concentration) at 20 °C and at 37 °C (Figure 1).

Out of all the components, xanthan had the highest intrinsic viscosity which decreased with increase in temperature. This could be due to conformational transition between rigid to a disordered flexible state [31]. There is a high amount of error in 37 °C sample meaning that this trend is not statistically significant (*p* = 0.41). However, similar behavior in specific viscosity of xanthan was found in a related study [32]. We observed a critical overlap point [33] for xanthan at 1.75mg/mL sample concentration and above. Therefore, the experiment was limited to 1.75 mg/mL. 

Intrinsic viscosity of alginate was close to the literature values [34] with slight differences due to the type of salt and buffer molarity. Increase in temperature did not result in any change in intrinsic viscosity of alginate. Alginate exhibited higher intrinsic viscosity measurement compared to degludec; however, it was less viscous than xanthan on its own. 

IDeg had the lowest intrinsic viscosity of all the components. Unlike the two polysaccharides, significant decrease in intrinsic viscosity of IDeg was observed with an increase in temperature (*p* = 0.022). This emphasises the fact that proteins and polysaccharides are structurally different, hence their solution properties are also different from each other. 

The binary system (XA) showed intrinsic viscosity profile midway between xanthan and alginate on their own. This could be attributed to the strong interactions among the two polysaccharides. Similar behaviour has been reported [10]. Increase in temperature resulted in a significant decrease in intrinsic viscosity of XA mixture (*p* < 0.05). This coincides with the trend observed from the individual polysaccharide behaviour, but more pronounced, suggesting a strong interaction.

PIC appeared to be less viscous than XA. Nevertheless, intrinsic viscosity of XA and PIC lie perfectly in the centre of the individual components which means it is approximately an average of the participating components. Contrary to the single and binary components, intrinsic viscosity of PIC significantly increased with an increase in temperature (*p* < 0.05). This indicates that X and A in the presence of IDeg are behaving differently compared to the binary system of XA and the single component systems. To elucidate these changes further, other investigative methods were employed. Overall, the intrinsic viscosity trend was as follows, X > XA > PIC > A > IDeg (Supplementary Table S2).

### 3.2. Particle Size Distribution 

Particle size distribution (PSD) was used to assess the size distribution, aggregation or dissociation of the polymers under investigation based on their diffusion properties. 

**Figure 2:**
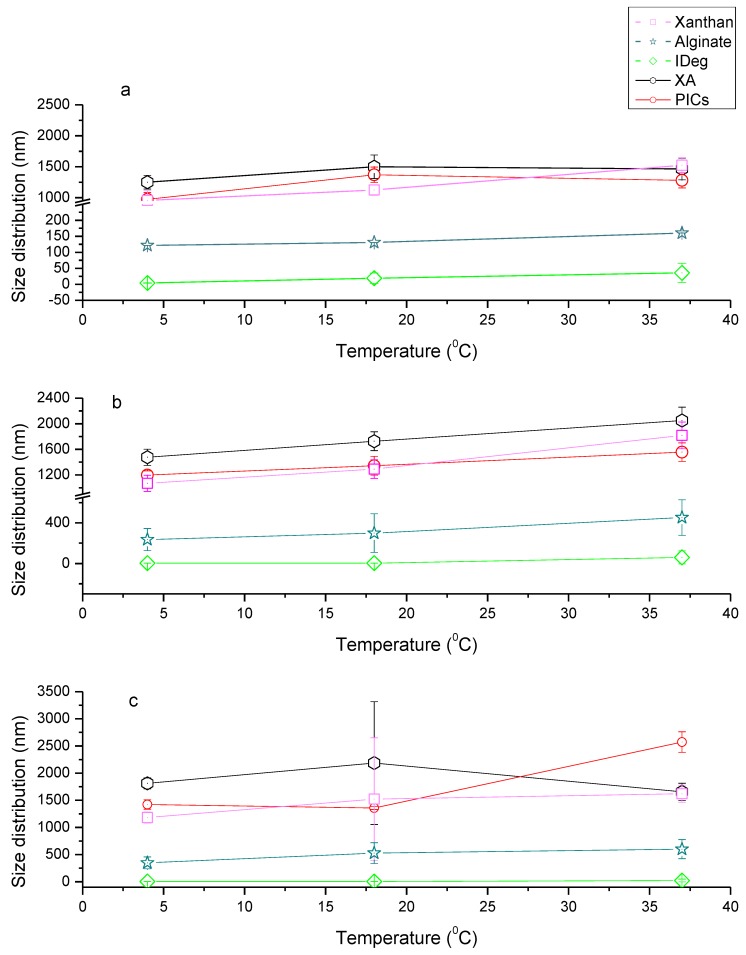
Particle size distribution of Xanthan (X), alginate (A), Insulin degludec (IDeg), XA, and PIC at a range of temperatures (4, 18 and 37 °C) on (**a**) Day 1, (**b**) Day 7 and (**c**) Day 14.

Of each single component, xanthan had the highest PSD (Figure 2), which is consistent with findings from intrinsic viscosity measurement. There was an increase, though insignificant, in PSD of xanthan with an increase in temperature and time (F_1,2_ = 33.50, *p* = 0.11) which was higher than what was reported previously [35]. From Day 1 to Day 7 and Day 14, PSD of xanthan significantly increased on Day 14 (F_1,2_ = 301.41, *p* = 0.03) but not on Day 7 (F_1,2_ = 35.49, *p* = 0.11) (Figure 2, Supplementary Table S3). Overall, PSD values for xanthan were between 956 and 1818 nm (radius). Conformation of xanthan is known to be stable at lower temperatures and remain stable up to 80 °C, maintaining its viscosity at pH 7 [36]. However, xanthan can also adopt a transitional conformation change above 40 °C, which is described as order-disorder state of helical conformation [37]. In our study, it is possible that at 37 °C this process would have initiated, which resulted in an increase in PSD. 

Alginate was the second biggest single component with PSD of 93–588 nm radius (Figure 2). PSD for alginate appeared to increase with temperature, albeit insignificant (*p* = 0.12). Additionally, there was no significant increase in PSD of alginate at all the three time points, on Day 1 (F_1,2_ = 28.42, *p* = 0.12), Day 7 (F_1,2_ = 60.45, *p* = 0.08) and Day 14 (F_1,2_ = 14.31, *p* = 0.16). 

PSD for IDeg at 4 °C was approximately 4 nm, which is in agreement with previous studies [38,39]. On Day 1, significant changes were observed in IDeg when it was subjected to increasing temperature (F_1,2_= 375.01, *p* = 0.03). Although there was a trend showing increase in PSD for IDeg at corresponding temperatures, these changes were not significant over time (Day 7- F_1,2_= 25.17, *p* = 0.13 and Day 14- F_1,2_ = 1.16, *p* = 0.48). It is posited that conformational change of IDeg from di-hexameric to multi-hexameric state may account for this increase in PSD [40]. Jonassen et al [40] has described in-vivo phenol depletion to be responsible for this conformational change. As the current investigation was performed on insulin containing phenol, this result indicates physiological temperature plays a role in insulin conformational change.

The PSD for binary system (XA) at 40C on Day 1, was found to be ~1251 nm. This was higher than both polysaccharides on their own, which reflects conformational changes in the structure of the macromolecules. Increase in temperature on Day 1 had an insignificant impact on PSD of XA (F_1,2_ = 2.44, *p* = 0.36). However, when incubation time was increased, significant increase in size was observed with an increase in temperature, Day 7 (F_1,2_ = 4382.43, *p* = 0.01). These changes in PSD were stable as no significant increase in PSD was observed from day 7 to 14 (F_1,2_ = 4.52, *p* = 0.28). 

PSD of the PIC were also found to be almost an average of PSD of single components. PIC had a gradual increase in PSD with an increase in temperature and incubation time. Significant changes were observed on day 7 with an increase in temperature (F_1,2_ = 3395.92, *p* = 0.01), respectively. However, further incubation time and increase in temperature did not lead to significant changes in PSD of PIC (F_1,2_ = 0.29, *p* = 0.68) (Supplementary Table S3).

Overall PSD trend was X > A > IDeg > XA > PIC which is in contrary to the intrinsic viscosity outcome. This could be due to the fact that viscosity measurements were taken in limited conditions. Nevertheless, it was observed that PSD at 20 °C and 37 °C on day 1 coincide well with intrinsic viscosity outcome. 

### 3.3. Zeta (ζ) Potential Analysis

Electrophoretic mobility or zeta (ζ) potential analysis was used to identify the strength of interactions among the components of the complex.

The ζ-potential is influenced by the biochemical nature of the surrounding aqueous solution. This involves the concentration, type of salt and, fundamentally, the pH [41,42]. ζ-potential values between +30 mV to −30 mV mean particles are unstable and coalesce. A higher ζ-potential means higher electrostatic repulsion which in turn means less aggregation and highly stable colloidal system [43]. The functional groups present on the surface of the molecule can dissociate or can be adsorbed to the ions present in the surrounding solution. 

**Figure 3:**
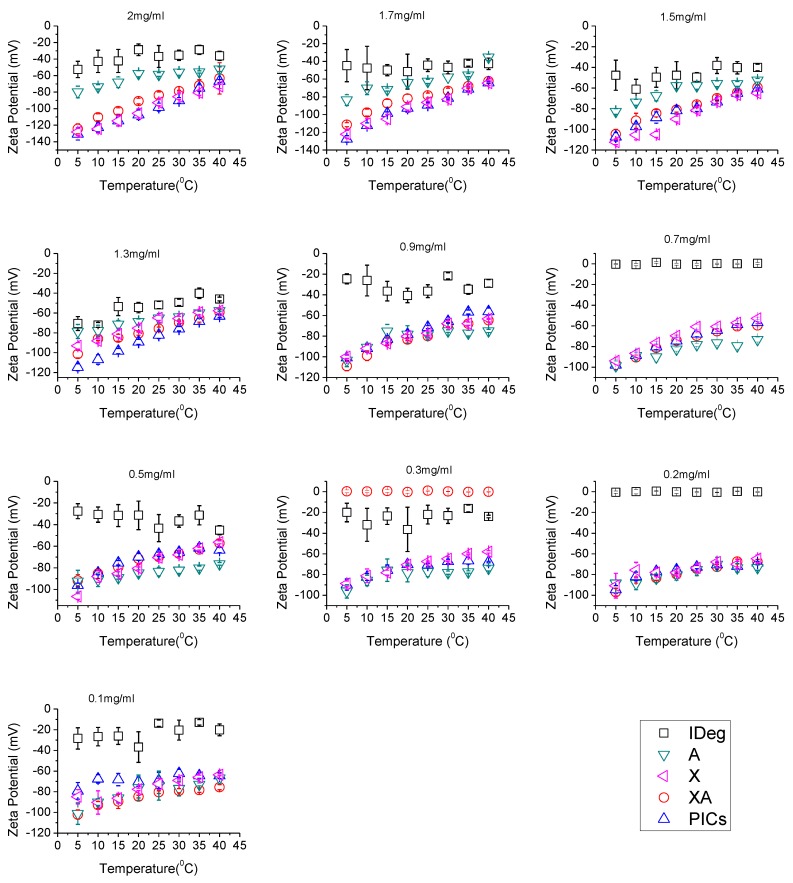
Zeta potential analysis of the Xanthan, Alginate, IDeg mixture of xanthan and alginate and PIC at a range of concentrations (0.1–2.0 mg/mL in 50 mM PBS, pH 7.0) and temperatures (5–40 °C).

ζ-potential was measured for all components at a range of concentrations (0.1–2.0 mg/mL) and temperature (5 °C to 40 °C). The general trend of ζ-potential was IDeg < A < XA < X ≈ PIC. For concentrations above 1.3 mg/mL, these changes were distinctive. Any concentration below this gave overlapping results (Figure 3). It was also observed that all macromolecules on their own and as a complex presented more negative surface charge at a lower temperature (5 °C). Increase in temperature resulted in a decrease in the ζ-potential values which refers to the conformational changes, agglomeration and possible interactions [12]. 

It was observed that, of the single components, IDeg had the highest (least negative) surface charge of approximately between −40 mV to −65 mV at 5 °C higher concentrations (from 2 to 1.3 mg/mL). Further reduction in concentration resulted in the fluctuations of ζ values for IDeg. Increase in temperature resulted in a decrease in ζ-potential values of IDeg. However, this difference became minimal at lower concentrations (Figure 3).

Alginate surface charge values from 2.0 mg/mL to 1.3 mg/mL were found between −50 mV to −80 mV. Further reduction in concentration resulted in an increase in alginate ζ-potential. Moreover, in all concentrations, increase in temperature resulted in a decrease in ζ-potential values for alginate. 

Xanthan followed the parallel trend for ζ-potential values as alginate but xanthan appeared to have higher ζ-potential than alginate. Above 1.3 mg/mL, ζ-potentials between −70 mV to −130 mV were obtained. Further reduction in concentration of both polysaccharides yielded similar surface charges (Figure 3). Likewise, increase in temperature decreased ζ-potential for xanthan which implies structural changes [31].

The mixture of XA displayed more negative surface charges than alginate on its own and less negative than xanthan on its own. PIC had ζ-potential values very close to the xanthan on its own.

IDeg in a pharmaceutical formulation exists as a di-hexamer with only one pole of the structure exposing positive charge of zinc ion, involved in di-hexamer formation [40]. Upon injection, in a physiological environment, this di-hexamer dissociates and changes conformation allowing Zn^2+^ and amino acids located within to interact with other IDeg di-hexamers and to form long multi-hexamer chains in the subcutaneous tissue [44]. It is therefore possible that preparation of PIC dilutions in PBS buffer, resulted in the depletion of phenol and allowed certain conformational changes in IDeg that resulted in its interaction with both polysaccharides at low temperature. At 37 °C (physiological temperature), it is thought that the complex released the insulin which formed multi-hexamers which may account for the reduction in ζ-potential values as observed.

Furthermore, the PIC had surface charge values in the region of surface charge values of xanthan on its own, which may lead to the conclusion that xanthan has more influence on the overall interactions among all other components of the complex. 

Statistically, zeta potential was significantly influenced by changes in all parameters (sample type, concentration, and temperature) (Supplementary Table S4). It means that changes in one of them will influence the surface charge, interactions, as well as the process of complexation. 

### 3.4. Sedimentation Velocity 

In order to identify the interactions and stability of the PIC constituents, sedimentation velocity experiments were performed. Single and double component systems were used in order to establish a baseline for the sedimentation properties of each constituent while the tri-component system represented the PIC. The ls-g*(s) method in SEDFIT was used to obtain sedimentation coefficient distributions. Concentration of peak was measured by area under curve (AUC) using Origin Pro 8. Any AUC value for double and triple component systems, less than the sum of single component AUC values, was considered as interaction among the component or vice versa. If AUC values of double and triple component system were higher than the sum of the single component AUC values, it was considered as no interaction. 

**Figure 4:**
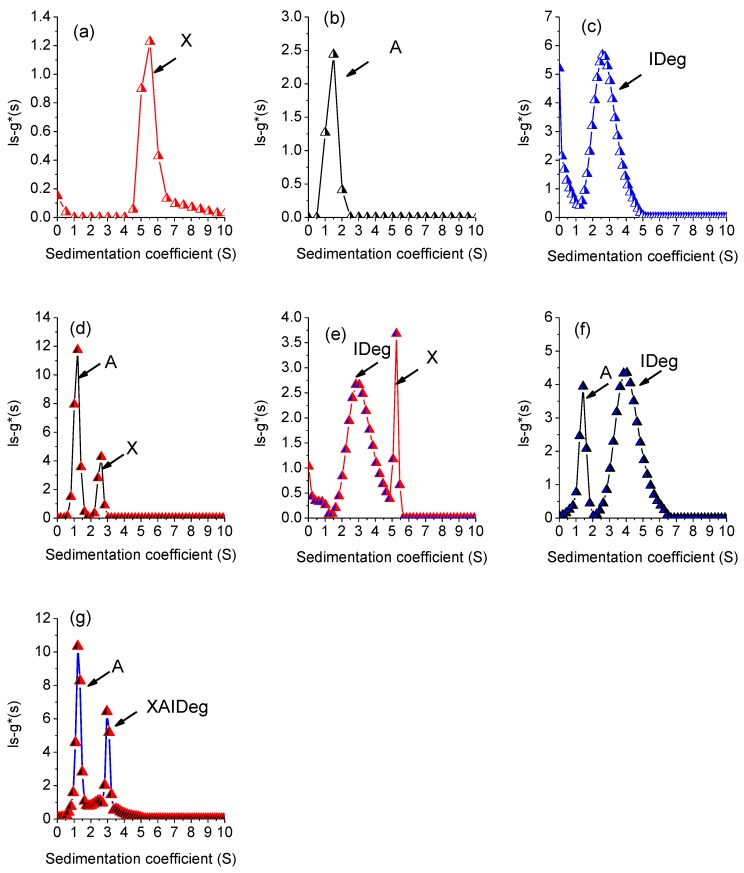
Plots of sedimentation coefficient profiles for single, double and triple-component system where (**a**) xanthan, (**b**) alginate (**c**) Insulin Degludec, (**d**) X + A, (**e**) X + IDeg, (**f**) A + IDeg, (**g**) PIC = X + A + IDeg.

Broad peaks were obtained for all single component system (Figure 4). However, s^0^_20w_ for xanthan was calculated to be 5.5S (Figure 4a) which is lower than 12.97S as reported by Dhami et al. 1995 [45] and higher than 3.5S as reported by Abdelhameed et al. 2010 [46]. Sedimentation properties are highly dependent on the solution conformation of the macromolecule. Xanthan can interchange its conformation between double helix to single stranded and coil conformation based on the ionic strength of the solution [47,48], which explains difference in s^0^_20w_ for xanthan in different studies (A concentration series was performed but data not shown). The s^0^_20w_ for alginate (Figure 4b) were similar to the values as reported previously [46]. The respective sedimentation coefficient values for all single components are given in Table 1. 

IDeg (Figure 4c) sedimented at 2.6S which is lower than the values reported earlier [39,49]. Sedimentation properties are greatly influenced by the ionic strength of the solvent [50,51]. Use of a lower ionic strength buffer (50 mM PBS) could be the reason for lower apparent sedimentation coefficient of IDeg [39].

Sedimentation behaviour of the two-component system was different from individual macromolecules. The mixture of the two polysaccharides (X + A) revealed a decrease in the sedimentation coefficient values for both components (Figure 4d, Table 2). This decrease in sedimentation coefficients suggests that the two polymers had an impact on each other’s sedimentation properties (co-sedimentation effect) [52] that could possibly include change in conformation. Furthermore, there was an increase in the total area under curve (AUC) for both peaks (Table 2), which indicates no interactions between the two polymers besides having an influence on sedimentation behaviour.

In the two-component system of X + IDeg and A + IDeg, two peaks with a shift in sedimentation values were detected (Figure 4e,f). AUC for X + IDeg system came out to be lower than the individual component (Table 2). However, the two prominent peaks were also present with S-values very close to the individual components and very low sum of concentration indicates loss of material. Therefore, it is possible that difference in concentration could possibly be due to earlier sedimentation of high molecular weight components in the beginning of the experiment and not due to complex formation.

The three-component system was tested for the interactions of three components (X, A and IDeg) to form a complex (PIC) (Figure 4g). Based on the sedimentation values (Table 3), it was concluded that first peak at 1.21S is for alginate and peak 2 at 2.96 is for the complex PIC. It is possible that due to complex formation and co-sedimentation a segment of alginate sedimented at 1.21 instead of 1.5 and remaining alginate along with xanthan and IDeg made a complex. 

**Figure 5:**
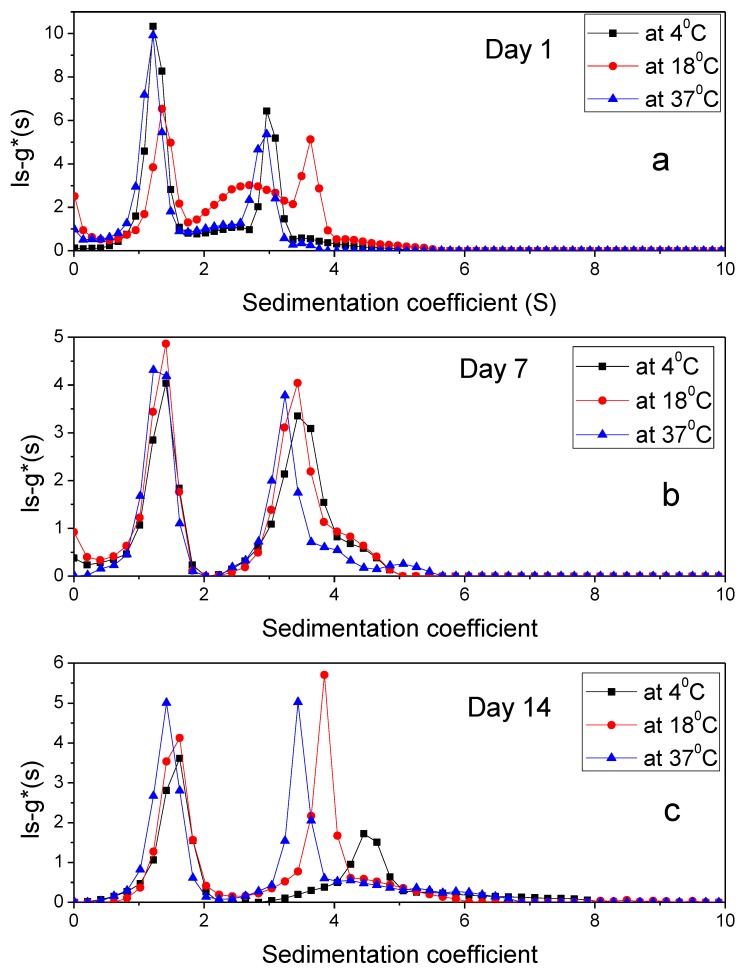
The sedimentation coefficient profile, ls-g*(s) for PIC (**a**) Day 1 (**b**) Day 7 (**c**) Day 14 using 50 mM PBS, pH 7.0 at 1 mg/mL loading concentration and at 4 °C, 18 °C, 37 °C.

Stability of the PIC was tested for 14 days under three different temperatures (Figure 5). 

AUC values (Table 3) indicated that there were interactions leading to stable complex formation between xanthan, alginate and IDeg. Expected values of the complex were calculated by summation of AUC values of individual polymers, which were 13.29 fringe units for PIC. Although, there was a shift in sedimentation coefficient values of the peaks over the period of two weeks and temperature of incubation (Table 3), the number of peaks remained constant, with an exception at 37 °C on day 7 for PIC complex. Therefore, it was concluded that interactions among PIC component were stable and the complex did not disintegrate at lower temperature for the tested timeframe (day 1–14). It is possible that at 37 °C the complex either dissociated or microbial growth would have occurred.

## 4. Conclusions

Protein–polysaccharide complexes have significant importance in the preparation of pharmaceutical formulations. We have investigated complexation of clinically relevant insulin degludec with alginate and xanthan in the absence of a linking polymer.

Biophysical methods were used to probe the complex behavior at different time points and temperatures. At lower temperatures (4 °C and 18 °C), interactions and complex formation were observed among the participating component as indicated by the sedimentation velocity plots (reduction in area under the curve). Moreover, the ζ-potential of PIC was highest among the all component which indicates highly stable system. PSD and ζ-potential values of PIC were close to xanthan on its own, which also indicates that xanthan is the most dominating component in the system which would possibly have a big influence on the interactions. Increase in PSD and the presence of three peaks (Table 3, day 7 at 37 °C) could possibly be due to the disintegration of complex or microbial growth. 

The complexation of insulin with alginate and xanthan can be a new area of investigation for formulation preparations and drug delivery for a plethora of health problems including diabetes.

## Figures and Tables

**Table 1:** Sedimentation coefficient (±SD, obtained from linear regression of s against c) of Alginate (1 mg/mL), xanthan (1 mg/mL) and Insulin (3.6 mg/mL) at pH 7.0, 50 mM PBS after incubation at 4 °C for 1 day. Analysis performed at 20 °C.

Polymer	Sedimentation Coefficient (S)	Area Under Curve (AUC)
Alginate	1.5 ± 0.016	2.09
Xanthan	5.5 ± 0.310	1.35
IDeg	2.6 ± 0.002	9.85

**Table 2:** Sedimentation coefficient for two component system of Alginate, Xanthan with IDeg at pH 7.0, 50 mM PBS at 4 °C. Expected values of area under curve for the two-component system are also given below. Any value below this means stable complex formation. ± values represent SD.

Mixture		Experimental AUC Values			Interaction Status
	Expected AUC	Identity	Sedimentation Coefficient (S)	AUC (Fringe Units)	
A + X	3.44	A	1.0 ± 0.003	4.18	S value decreased for both, 1.5 to 1S for A and 5.5 to 2.5S for X. No interactions due to co-sedimentation
		X	2.5 ± 0.002	1.11	
X + IDeg	11.20	X	5.25 ± 0.001	0.91	S value for IDeg change from 2.6S to 2.83S and for xanthan from 5.5S to 5.25S. No complex formation as sum of the two components AUC is very low. Earlier sedimentation
		IDeg	2.83 ± 0.003	4.79	
A + IDeg	11.94	A	1.42 ± 0.003	1.93	S value for Alginate decreased (from 1.5 to 1.42S) and Insulin increased (from 2.6 to 4.04S). Reduction in AUC for both polymers. Some interactions
		IDeg	4.04 ± 0.001	7.84	

**Table 3:** Sedimentation coefficient for PIC at pH 7.0, 50mM PBS at 4°C. Expected values area under curve for the peaks of X, A and IDeg Ʃ = 13.29, any value below 13.29 means stable complex formation.

	4 °C			18 °C			37 °C		
Days	Sedimentation Coefficient (S)	AUC (Fringe Units)	Interaction Status	Sedimentation Coefficient (S)	AUC (Fringe Units)	Interaction Status	Sedimentation Coefficient (S)	AUC (Fringe Units)	Interaction Status
**1**	1.21 ± 0.002 2.96 ± 0.001	3.69 2.05	Interaction occurred. A part of alginate sedimented separately and PIC formation occurred	1.39 ± 0.002 2.66 ± 0.007	3.77 1.54	Interaction occurred. A part of alginate sedimented separately and PIC formation occurred	1.32 ± 0.002 3.25 ± 0.007	2.029 2.605	Interaction occurred. A part of alginate sedimented separately and PIC formation occurred
**7**	1.41 ± 0.004 3.43 ± 0.004	1.89 6.212		1.46 ± 0.003 4.03 ± 0.002	2.56 8.64		1.26 ± 0.003 3.34 ± 0.002 5.00 ± 0.015	2.35 1.17 1.17	Some interactions may have occurred but most of the components remained separated
**14**	1.6 ± 0.004 4.3 ± 0.003	3.76 2.198		1.48 ± 0.002 3.85 ± 0.002	2.43 8.53		1.37 ± 0.003 3.65 ± 0.004	2.32 1.75	Some interactions may have occurred but most of the components remained separated and sedimented quickly, hence only two peaks were visible

## Supplementary Materials

The following are available online at https://www.mdpi.com/2073-4360/12/2/390/s1, Table S1: Molecular weight and hydrodynamic radius of Xanthan, Alginate and IDeg, Table S2: Intrinsic viscosity (ml/g) of insulin degludec (IDeg), xanthan (X), alginate (A), Binary system containing xanthan and alginate (XA) and PIC measured at 20 °C and 37 °C, Table S3: Results for ANOVA test for PSD of Xanthan (X), alginate (A), Insulin degludec (IDeg), XA, and PIC at a range of temperatures (4, 18 and 37 °C) on Day 1, Day 7, and Day 14, Table S4: MANOVA analysis.

## Abbreviations

A	Alginate
X	Xanthan
IDeg	Insulin degludec
XA	Mixture of xanthan and alginate
PBS	Phosphate Buffer Saline
PIC	Polymeric insulin complex
PPC	Protein–polysaccharide complexes
s^0^_20w_	Sedimentation coefficient, corrected for non-ideality, temperature and buffer conditions (1S = 1Svedberg = 1 × 10^−13^ sec).
