# Supplementary material for: Clinically Relevant Insulin Degludec and Its Interaction with Polysaccharides: A Biophysical Examination
Source: Polymers (Basel). 2020 Feb 9;12(2):390. doi: 10.3390/polym12020390 (PMC7077624; doi:10.3390/polym12020390)
Supplement: Supplementary file 1 [file polymers-12-00390-s001.pdf]

Supplementary Table S1: Molecular weight and hydrodynamic radius of Xanthan, Alginate and IDeg

	Molecular Weight (kDa)	Hydrodynamic radius*[2] at 20°	Hydrodynamic radius*[2] at 37°
Xanthan	261	79	75
Alginate	56	15	15
IDeg	73*[1]	5	4.4

*[1]- Molecular weight of a dodecamer of insulin

*[2] –Calculated using $R_{h=}\left(\frac{3[\eta]M}{10\pi N}\right)^{1/3}$

Armstrong, J.K., et al., *The hydrodynamic radii of macromolecules and their effect on red blood cell aggregation.* Biophysical journal, 2004. **87**(6): p. 4259-4270.

Supplementary Table S2: Intrinsic viscosity (ml/g) of insulin degludec (IDeg), xanthan (X), alginate (A), Binary system containing xanthan and alginate (XA) and PIC measured at 20^{0}C and 37^{0}C

	**Intrinsic viscosity at 20^{0}C**	**R^2**	**Intrinsic viscosity at 37^{0}C**	**R^2**	**P value**
**X**	12000 ± 520	0.987	10300 ± 6000	0.968	0.55
**A**	360 ± 31	0.996	360±11	0.999	0.41
**IDeg**	9.2 ± 1.4	0.950	7.4 ±1.8	0.947	0.022
**XA**	1000 ± 160	0.976	590 ± 60	0.996	$3.8x10^{-13}$
**PIC**	490 ± 60	0.998	710 ± 58	0.998	$1.2x10^{-13}$

Supplementary Table S3: Results for ANOVA test for PSD of Xanthan (X), alginate (A), Insulin degludec (IDeg), XA, and PIC at a range of temperatures (4, 18 and 37^{0}C) on Day 1, Day 7, and Day 14

	Day 1		Day 7		Day 14	
**X**	$F_{1,2}$=33.50	P=0.11	$F_{1,2}$=35.49	P=0.11	$F_{1,2}$=301.42	P=0.04
**A**	$F_{1,2}$=28.42	P=0.12	$F_{1,2}$=60.45	P=0.08	$F_{1,2}$=14.31	P=0.16
**IDeg**	$F_{1,2}$=375.01	P=0.03	$F_{1,2}$=25.17	P=0.13	$F_{1,2}$=1.16	P=0.48
**XA**	$F_{1,2}$= 2.44	P=0.36	$F_{1,2}$=4382.43	P=0.01	$F_{1,2}$=4.52	P=0.28
**PIC**	$F_{1,2}$=1.07	P=0.49	$F_{1,2}$=3395.93	P=0.01	$F_{1,2}$=0.30	P=0.68

Supplementary Table S4: MANOVA analysis

Component	F statistic	Significance
**Sample**	$F_{4,1765}$ =6543	P<0.001
**Concentration**	$F_{10,1765}$ =487	P<0.001
**Temperature**	$F_{7,1765}$ =1181	P<0.001
**Sample*Concentration**	$F_{39,1765}$ =354	P<0.001
**Sample*Temperature**	$F_{28,1765}$ =51	P<0.001
**Temperature*Concentration**	$F_{70,1765}$ =13	P<0.001
**Sample*Temperature*Concentration**	$F_{273,1765}$ =4	P<0.001
